# Carcinomatous meningitis in a patient with Her2/*neu* expressing bladder cancer following trastuzumab and chemotherapy: a case report and review of the literature

**DOI:** 10.4076/1752-1947-3-9110

**Published:** 2009-09-15

**Authors:** Oscar B Goodman, Matthew I Milowsky, Jodi Kaplan, Maha Hussain, David M Nanus

**Affiliations:** 1 Division of Clinical Oncology, Nevada Cancer Institute, Las Vegas, NV 89135, USA; 2 Division of Hematology and Medical Oncology, Department of Medicine, Weill Medical College of Cornell University – New York Presbyterian Hospital, New York, NY 10021, USA; 3 Division of Hematology and Medical Oncology, Department of Medicine, University of Michigan, Ann Arbor, MI 48109, USA

## Abstract

**Introduction:**

Targeted therapies may impact the natural history of bladder cancer based upon their pharmacokinetics. The Her2/*neu* receptor tyrosine kinase, overexpressed by half of all primary urothelial carcinomas, has recently been examined as a therapeutic target in bladder cancer in a prospective phase II multicenter trial (NCI-198) that enrolled 109 patients with advanced bladder carcinomas for treatment with trastuzumab in combination with paclitaxel, carboplatin, and gemcitabine. We report on documented isolated Her2/*neu* positive carcinomatous meningitis in a patient treated with trastuzumab.

**Case presentation:**

A 61-year-old Caucasian man with metastatic bladder cancer was treated with neoadjuvant chemotherapy in combination with trastuzumab with a partial response that was followed by a complete response after surgery. He relapsed with isolated Her2/*neu* positive carcinomatous meningitis.

**Conclusion:**

Carcinomatous meningitis in bladder cancer is extremely rare. This is the first case reported of Her2/*neu* positive carcinomatous meningitis. Disease recurred solely at a sanctuary site, demonstrating that despite the systemic efficacy of trastuzumab in combination with chemotherapy, its inability to enter the central nervous system potentially contributes to the unusual site of disease recurrence.

## Introduction

The Her2/*neu* receptor tyrosine kinase is overexpressed by the majority of all primary invasive urothelial carcinomas [1]. The epidermal growth factor receptor (EGFR) tyrosine kinase family comprises four members (erbB-1 through erbB-4), with erbB-1 (EGFR) and erbB-2 (Her2/*neu*) expressed in urothelial carcinoma [2]. Following ligand activation, the receptors dimerize resulting in stimulation of multiple signaling pathways, leading to increased cell growth and survival [3]. Her2/*neu-*mediated signaling activates important oncogenic signaling cascades such as the ras-mitogen activated protein (MAP)-kinase pathway, phospholipase C-gamma (PLC-γ) and phosphatidylinositol-3 (PI-3) kinase [4]. Overexpression of Her2/*neu* is associated with higher tumor grade and decreased disease-related survival [5], suggesting a specific role for Her2/*neu* in bladder cancer progression. Gene amplification is rarely observed, in contrast to breast cancer where gene amplification is seen in about 25% of cases and correlates with Her2/*neu* protein overexpression [6]. Although the mechanism for gene overexpression is not well understood, most evidence points to a transcriptional mechanism mediated by the transcription factor OB2-1 [7]. We report the case of a patient who had a complete response to surgery but relapsed with isolated Her2/*neu* positive carcinomatous meningitis. Advances in multimodality therapies including neoadjuvant chemotherapy in bladder cancer may alter the natural history of this disease.

This case represents the first report of Her2/*neu* positive urothelial carcinomatous meningitis. Several recent studies have implicated Her2/*neu* overexpression in the progression of urothelial carcinoma. Overexpression of Her2/*neu* is associated with higher tumor grade and decreased disease-related survival [5]. A cohort study of 245 patients revealed that 45% of the tumors expressed Her2/*neu* protein and expression correlated with higher grade, tumor recurrence, and decreased survival, especially when co-expressed with ErbB1 or ErbB3 [8]. In a series of 80 consecutive cases of muscle-invasive urothelial bladder carcinomas, Jimenez and colleagues showed that 45% of Her2/*neu* negative primary disease had Her2/*neu* positive metastatic nodal disease, while only one case (8%) of Her2/*neu* positive primary disease manifested with Her-2/*neu* negative nodal metastatic disease [9]. Collectively, these data indicate that Her2/*neu* expression may be predictive of tumor aggressiveness and contribute to metastasis.

The phase II NCI-198 (NCT00005831) trial prospectively evaluated the safety and efficacy of open label trastuzumab in combination with chemotherapy in patients with documented Her2/*neu* positive advanced urothelial carcinoma. Eligible patients received paclitaxel (200 mg/m^2^ day 1), carboplatin (AUC 5 day 1), gemcitabine (800 mg/m^2^ days 1, 8) and trastuzumab (4 mg/kg loading dose, then 2 mg/kg days 1, 8, 15) every 21 days. Of 109 patients screened for the study, 57 (52%) were Her2/*neu* positive and of these, 44 were eligible for protocol therapy. Her2/*neu* positive patients had a greater mean number of metastatic sites (2 versus 1, p = 0.014). The overall response rate was 31/44 (70%), with a median time to progression of 9.3 months and a median survival of 14.1 months [10]. These findings compared favorably with historical controls, for example, gemcitabine/cisplatin-treated patients had an overall response rate of 49%, a median progression-free survival of 7.7 months and a median survival of 14.0 months [11,12]. Notably, however, nearly one-third of these patients had tumors that were not metastatic, possibly explaining the similar median survival between the two groups [10].

## Case presentation

A 61-year-old Caucasian man with a history of benzidine exposure and tobacco use presented with intermittent gross hematuria over the previous 2 months. Cystoscopy and transurethral resection of the bladder revealed a 2 cm high grade muscle invasive urothelial carcinoma. A staging computed tomography (CT) scan identified extensive retroperitoneal lymphadenopathy with a conglomerate of nodes at the aortic bifurcation measuring 7.6 × 3.5 cm and with the largest individual node found at the level of the right common iliac bifurcation measuring 2.7 × 3.9 cm. Biopsy of this lymph node confirmed metastatic urothelial carcinoma. Immunohistochemical analysis of the primary tumor revealed 3+ Her2/*neu* positivity, while fluorescence *in situ* hybridization (FISH) analysis revealed no Her2/*neu* gene amplification. The patient was enrolled on the NCI-198 trial with paclitaxel (200 mg/m^2^ day 1), carboplatin (AUC 5 day 1), gemcitabine (800 mg/m^2^ days 1, 8) and trastuzumab (4 mg/kg loading dose, then 2 mg/kg days 1, 8, 15) every 21 days. After six cycles, a CT scan demonstrated a partial response in the retroperitoneum, with the right common iliac node measuring 1.2 × 0.8 cm, corresponding to a >95% decrease in volume [13]. He then underwent a radical cystectomy and extensive lymph node dissection with removal of 69 nodes, revealing pT2aN0M0 high grade disease and resulting in a surgically rendered complete response.

**Figure 1:**
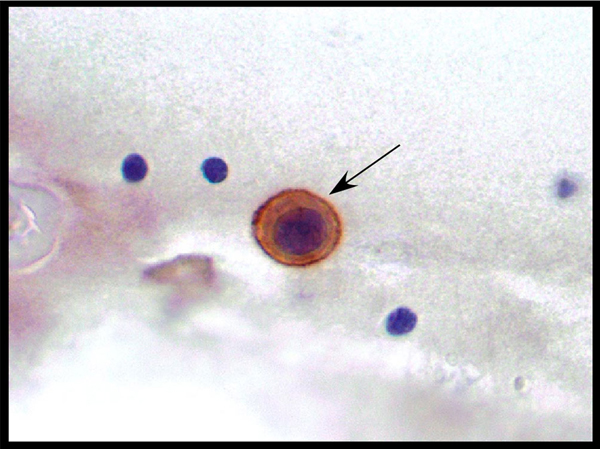
**Immunohistochemical staining of cerebrospinal fluid revealing a 2+ Her2/*neu* positive malignant cell (arrow)**.

**Figure 2:**
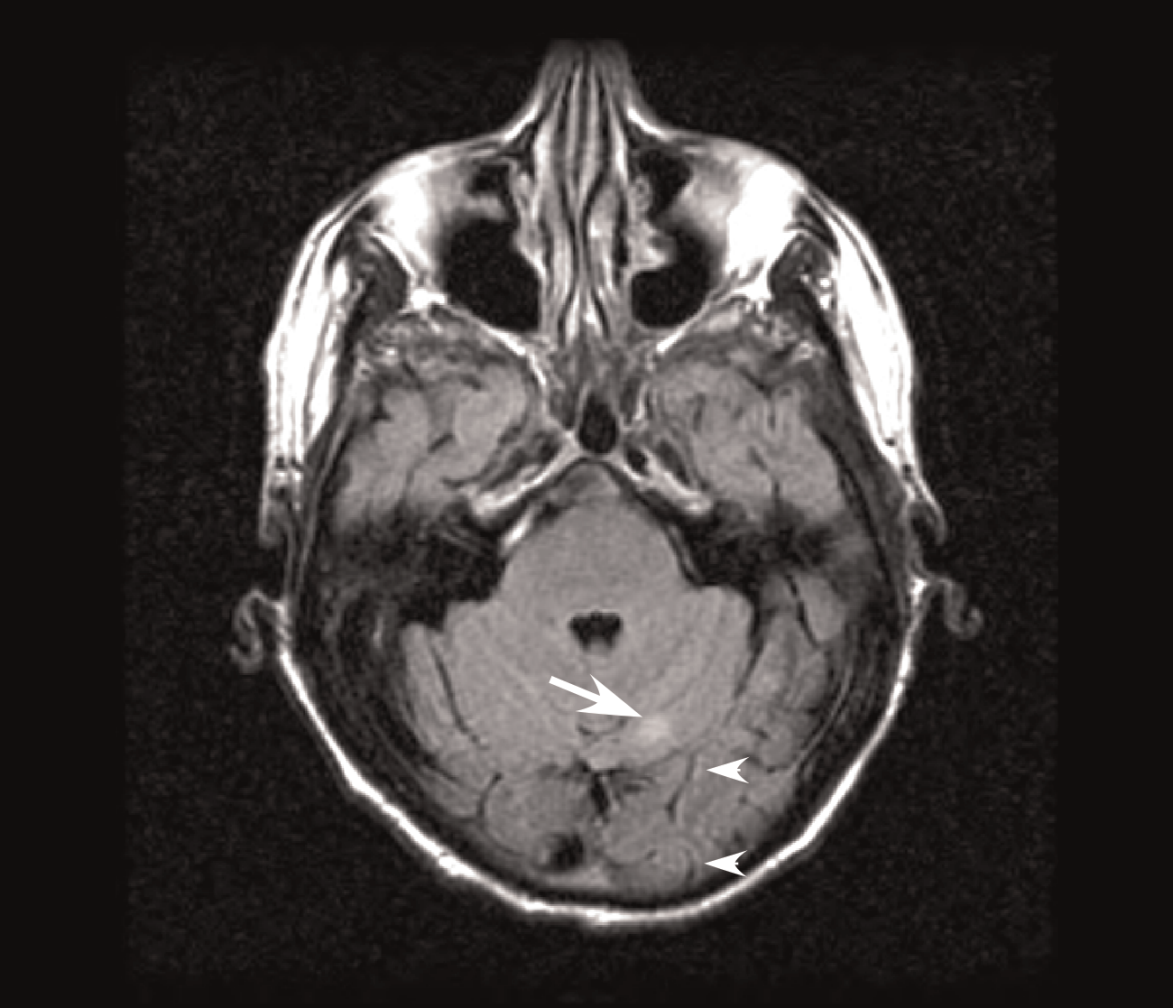
**T1-weighted brain magnetic resonance imaging demonstrating both leptomeningeal enhancement (arrowheads) and a cerebellar metastasis (arrow)**.

Two weeks postoperatively, the patient developed paresthesia and proprioceptive deficits in his hands and feet that were attributed to nerve compression that occurred during prolonged surgery. Nerve conduction studies revealed an axonal sensorimotor polyradicular neuropathy with demyelinating features. Neurologic symptoms did not improve with gabapentin therapy and a lumbar puncture five months postoperatively revealed carcinoma cells. Immunohistochemical analysis of the cerebrospinal fluid (CSF) revealed 2+ Her2/*neu* expression (Figure 1). Magnetic resonance imaging (MRI) of the brain confirmed diffuse leptomeningeal enhancement along with cerebellar metastases (Figure 2). A CT scan of the chest, abdomen and pelvis revealed no evidence of systemic recurrence. An Ommaya reservoir was placed and the patient received four weeks of biweekly intrathecal methotrexate therapy, resulting in negative repeat cytologic CSF evaluations after two weeks of therapy. After week four, intrathecal therapy was stopped due to urosepsis, and a repeat cytologic evaluation one week later revealed recurrent carcinomatous meningitis. The patient refused further chemotherapy and died two weeks later.

## Discussion

Following treatment, our patient relapsed with isolated Her2/*neu* positive carcinomatous meningitis. Carcinomatous meningitis is rare in bladder cancer [14]-[16]. Trastuzumab does not cross the blood–brain barrier [17], suggesting that targeting Her2/*neu* positive systemic disease may alter the natural history of bladder carcinoma metastasis predisposing to the onset and progression of central nervous system (CNS) disease. Her2/*neu* expression in breast cancer CNS metastases is highly concordant with systemic disease status, with 93% of patients with Her2/*neu* positive primary tumors also expressing Her2/*neu* in CNS metastatic disease [18]. Given similar concordance in bladder cancer [9] as well as the fact that Her2/*neu* expression is more common in bladder cancer than in breast cancer [5], it remains to be seen if the incidence of carcinomatous meningitis due to leptomeningeal bladder metastases will increase in the setting of trastuzumab therapy as a consequence of systemic therapeutic efficacy and poor CNS bioavailability.

Despite the presence of Her2/*neu* positive disease, it is possible that the initial clinical response and subsequent CNS relapse in our patient was not due to trastuzumab but to carboplatin, gemcitabine and paclitaxel. Of the chemotherapeutic drugs, carboplatin has the highest CNS penetration with a peak CSF/plasma ratio of 28% but with significant interpatient variability (range 17-46%) [19], while gemcitabine and paclitaxel have markedly lower CNS penetration, with CSF:plasma ratios of 6.7% [20] and less than 1.8% (the limit of detection) [21], respectively. Thus, it is likely that the combination of paclitaxel, carboplatin and gemcitabine (TCG) has little if any therapeutic efficacy for CNS disease, while TCG in combination with trastuzumab may augment the systemic disease response, thereby predisposing to CNS relapse.

## Conclusions

Carcinomatous meningitis is exceedingly rare in bladder cancer, with only a few cases reported. Trastuzumab may alter the natural history of bladder carcinoma metastasis predisposing to CNS relapse. This likely reflects the potential efficacy of the therapy as well as an inability of chemobiologic therapy to penetrate the CNS. As a consequence of its systemic therapeutic efficacy and poor CNS bioavailability, trastuzumab may alter the natural history of bladder carcinoma resulting in an unusual presentation of metastatic disease.

## Abbreviations

CNS: central nervous system; CSF: cerebrospinal fluid; EGFR: epidermal growth factor receptor; FISH: fluorescence *in situ* hybridization; MAP: mitogen activated protein; MRI: magnetic resonance imaging; NCI: National Cancer Institute; PI-3: phosphatidylinositol-3; PLC-γ: phospholipase C-gamma; TCG: paclitaxel, carboplatin and gemcitabine.

## Consent

Written informed consent was obtained from the patient for publication of this case report and any accompanying images. A copy of the written consent is available for review by the Editor-in-Chief of this journal.

## Competing interests

The authors declare that they have no competing interests.

## Authors' contributions

OG assisted in the acquisition and analysis of the data, and in drafting the manuscript. MM analyzed the data and assisted in drafting the manuscript. JK assisted in the acquisition and analysis of the data. MH aided in the conception and design of this study, and in the acquisition and analysis of the data. DN assisted in the acquisition and analysis of the data, as well as drafting the manuscript. All authors have read and approved the final manuscript.
